# A Novel ZIP4-HDAC4-VEGFA Axis in High-Grade Serous Ovarian Cancer

**DOI:** 10.3390/cancers13153821

**Published:** 2021-07-29

**Authors:** Qipeng Fan, Lihong Li, Tian-Li Wang, Robert E. Emerson, Yan Xu

**Affiliations:** 1 Department of Obstetrics and Gynecology, Indiana University School of Medicine, 950 W. Walnut St. R2-E380, Indianapolis, IN 46202, USA; qifan@iu.edu; 2 Department of Gynecology and Obstetrics, Johns Hopkins Medical Institutions, 600 North Wolfe St., Baltimore, MD 21287, USA; lilihong1990@gmail.com; 3 Department of Gynecology, Oncology, and Pathology, Johns Hopkins Medical Institutions, 1550 Orleans Street, Baltimore, MD 21231, USA; tlw@jhmi.edu; 4 Department of Pathology and Laboratory Medicine, Indiana University School of Medicine, Indiana University Health Pathology Laboratory, 350 W. 11th Street, Room 4010, Indianapolis, IN 46202, USA; reemerso@iupui.edu

**Keywords:** cancer stem cell (CSC), histone deacetylase 4 (HDAC4), high-grade serous ovarian cancer (HGSOC), endothelial growth factor A (VEGFA), ZIP4

## Abstract

**Simple Summary:**

Despite tremendous research efforts, epithelial ovarian cancer (EOC) remains one of the most difficult cancers to detect early and treat successfully for >5-year survival. We have recently shown that ZIP4, a zinc transporter, is a novel cancer stem cell (CSC) marker and a therapeutic target for EOC. The current work focuses on developing new strategies to target ZIP4 and inhibit its CSC activities in EOC. We found that cells expressing high levels of ZIP4 were supersensitive to a group of inhibitors called HDACis. One of the major targets of these inhibitors is a protein called HDAC4. We revealed the new molecular bases for the ZIP4-HDAC4 axis and tested the efficacies of targeting this axis in the lab and in mouse models. Our study provides a new mechanistic-based targeting strategy for EOC.

**Abstract:**

We have recently identified ZIP4 as a novel cancer stem cell (CSC) marker in high-grade serous ovarian cancer (HGSOC). While it converts drug-resistance to cisplatin (CDDP), we unexpectedly found that ZIP4 induced sensitization of HGSOC cells to histone deacetylase inhibitors (HDACis). Mechanistically, ZIP4 selectively upregulated HDAC IIa HDACs, with little or no effect on HDACs in other classes. HDAC4 knockdown (KD) and LMK-235 inhibited spheroid formation in vitro and tumorigenesis in vivo, with hypoxia inducible factor-1 alpha (HIF1α) and endothelial growth factor A (VEGFA) as functional downstream mediators of HDAC4. Moreover, we found that ZIP4, HDAC4, and HIF1α were involved in regulating secreted VEGFA in HGSOC cells. Furthermore, we tested our hypothesis that co-targeting CSC via the ZIP4-HDAC4 axis and non-CSC using CDDP is necessary and highly effective by comparing the effects of ZIP4-knockout/KD, HDAC4-KD, and HDACis, in the presence or absence of CDDP on tumorigenesis in mouse models. Our results showed that the co-targeting strategy was highly effective. Finally, data from human HGSOC tissues showed that ZIP4 and HDAC4 were upregulated in a subset of recurrent tumors, justifying the clinical relevance of the study. In summary, our study provides a new mechanistic-based targeting strategy for HGSOC.

## 1. Introduction

Epithelial ovarian cancer (EOC) and high-grade serous ovarian cancer (HGSOC) in particular, has a poor 5-year survival rate [1,2,3,4]. Despite tremendous research efforts, ovarian cancer remains one of the most difficult cancers to detect early and treat successfully for >5-year survival [5]. The rates of mortality for ovarian cancer have been stagnant since around 1980 [6]. Hence, new therapeutic approaches based on cell signaling and mechanistic studies are urgently needed.

Most HGSOC cases become resistant to standard chemotherapy and are often refractory to additional therapeutic interventions [1,2,3,4]. Cancer stem cells (CSCs) or tumor-initiating cells are a small subset of tumor cells with tumor initiating capacity, which have been identified in leukemias and solid tumors, including EOC [7,8,9,10,11]. CSCs contribute directly to drug-resistance and are, in general, associated with multidrug resistance (MDR). In fact, side population, one of the standard methods to isolate CSC, detected by fluorescence-activated cell sorting (FACS) analyses for CSCs is a direct assay for high levels of efflux of the Hoechst33342 dye by ABC transporters reflecting MDR [12]. Hence, CSCs represent an important target for novel therapeutic strategies aimed at eradicating cancer. However, how to incorporate the targeting of CSCs into clinical practice is a major challenge for EOC and other cancers. In particular, HGSOC is one of the most heterogeneous among solid cancers [13], and so are its CSC markers. More than 10 EOC CSC markers have been identified, including side population, CD133, ALDH1/2, LY6A, LGR5, EpCAM, CD133, CD44, CD34, CD24, CD117, MyD88, and CDH1 [12,14]. In addition, the Wnt, SONIC Hedgehog, NOTCH, PI3K/PTEN, MAPK, and NF-κB signaling pathways have been implicated in EOC CSC [14]. Which one to target and how to target these cells effectively remain major challenges in the EOC field [8,14]. Cell signaling studies of CSC markers are crucial to move the field forward.

ZIP4 (gene name *SLC39A4*, a zinc transporter) plays tumor promoting roles in many cancer types, including pancreatic cancer, hepatocellular carcinoma, breast cancer, and glioma [15,16,17,18,19,20,21,22,23,24,25,26,27,28,29]. Most, if not all, of the cellular signaling and functions of ZIP4 have been linked to its zinc transporting activity. We have recently identified ZIP4 as a novel CSC marker and a new ZIP4-NOTCH3 axis as a novel target in HGSOC [16,17]. In addition, our data have shown that ZIP4 is also a powerful target for CSC, due to its upstream driver function in regulating several other CSC markers, its functional involvement in drug-resistance and sphere formation, as well as its potent tumor initiating capacity [17]. However, how a cell plasma membrane zinc transporter (not a transcription factor) transmits its cell signaling and functions (including regulating expression of other oncogenes) and which proteins are its important downstream mediators, in addition to its zinc transporting activity, are largely unknown.

Epigenetic modifications such as histone modification play an important role in tumorigenesis and in CSC in particular [30,31]. In humans, 18 histone deacetylase (HDAC) enzymes have been classified into four classes based on their homology with yeast HDACs, on the basis of size, sequence homology, as well as formation of distinct complexes [30,31,32]. Classes I, II, and IV require zinc as a cofactor in their active sites and are inhibited by zinc-binding HDAC inhibitors (HDACis). Class I HDACs consist of HDAC 1, 2, 3, and 8, which are ubiquitously expressed and predominantly located in nuclei. Class II HDACs exhibit a tissue specific pattern of expression, can be expressed in both nuclei and cytoplasm, and target both histone and nonhistone proteins [33]. In addition, Class II HDACs are further subdivided into Class IIa (HDACs 4, 5, 7, and 9), containing a ~600 amino acid N-terminal domain [30,32] and Class IIb (HDACs 6 and 10), characterized by having two deacetylase domains. HDAC11 is the only member of class IV [30,32].

Upregulation of HDACs have been reported in many cancers, including EOC [ref. [34,35,36], and cited articles herein]. In particular, the Class IIa HDAC4 is overexpressed in EOC and is correlated to poor overall survival and/or unfavorable progress-free survival in all ovarian cancer patients (*n* > 1400) examined [37,38]. HDAC4 is closely and functionally related to drug resistance [38,39,40,41,42,43,44,45]. Intriguingly, HDAC4 deacetylates and stabilizes HIF1α [46,47], one of the central players of tumor progression and drug response [48,49,50]. HDAC4 and HIF1α form a protein complex to regulate chemoresistance through protein phosphorylation, translocation and degradation in SKOV3 cells [38]. However, only minimal work has been conducted in linking HDAC4 and HIF1α and none of the work has linked their interaction to CSC.

HDACis are a family of synthetic and natural compounds that differ in their target specificities and activities. Several HDACis have been FDA approved for cancer treatment. In contrast to conventional chemotherapeutic agents, HDACis show strong tumor selectivity and cause less toxicity in normal tissues [51]. However, clinical trials with HDACi as single agents in solid tumors have been disappointing [52], indicating that combinational therapy is necessary for their clinical applications [30,53].

Many of the published and current studies focus on targeting CSC alone [reviews [31,54,55,56,57]) and references herein]. However, increasing lines of evidence suggest that co-targeting CSC and non-CSC cell populations in cancer treatment is important for the treatment of EOC. Yet, this type of study [36,57,58,59,60,61,62] is significantly underappreciated, partly due to a presumed and more rigid definition for CSCs, considering a cell population selected by a specific marker set as the only cell source initiating tumor formation. However, CSCs are highly dynamic and are interconvertible between CSCs and non-CSCs. Non-CSCs can be induced into a stem-like state enabling them to become drug-tolerant [12,63,64,65,66,67]. Hence, co-targeting both these subpopulations effectively is essential and further investigation is urgently needed to fill in the gaps before moving towards more effective targeting of CSC in clinics.

Upregulation of HIF1 stimulates CSCs via VEGF, Notch, and other signaling pathways [68]. More than 70 genes are HIF1 targets, with VEGFA as one of the best-characterized HIF1α targets [69,70,71]. VEGFA is highly elevated in ascites from human EOC patients, upregulated by lysophosphatidic acid, an oncolipid in EOC, and functionally involved in angiogenesis, tumor growth, metastasis, drug resistance, and vascular permeability [72,73,74,75,76,77,78,79,80,81,82,83]. In addition, bevacizumab, a humanized antivascular VEGF monoclonal antibody has been FDA-approved for EOC treatment [84,85,86,87]. It has been shown that ZIP4 overexpression causes significantly increased expression of several oncogenes, including VEGFA in both pancreatic cancer cell lines and xenografts [28]. However, the mechanisms of this upregulation are unknown.

Although combinational effects of an HDACi and CDDP in EOC cells have been tested (e.g., [36,59,60,61,62]), and reagents co-targeting CSC and non-CSC cell populations in cancer cell lines or mouse models have been reported (e.g., [57,58]), these studies did not test the effect in clearly defined CSC and non-CSC populations and were not linked to ZIP4. In the current work, we tested our hypothesis that co-targeting the ZIP4^+^ CSC (via ZIP4-knockout (KO)/knockdown (KD), HDAC4/KD, or HDACis) and the bulk ZIP4^−^ non-CSC (using CDDP) populations is highly effective to develop new treatment modalities for HGSOC. Instead of developing specific ZIP4 inhibiting reagents in the current work, we have developed an innovative approach to selectively and effectively target ZIP4 high cells, which mainly represent CSC as we have recently shown [17]. We then conducted mechanistic studies and revealed a novel ZIP4-HDAC4-VEGFA axis functionally involved in spheroid formation in vitro and tumorigenesis in vivo in HGSOC. Taken together, our study provides a new mechanistic-based targeting strategy for HGSOC.

## 2. Results

### 2.1. ZIP4 Sensitized HGSOC Cells to HDACis

**Figure 1:**
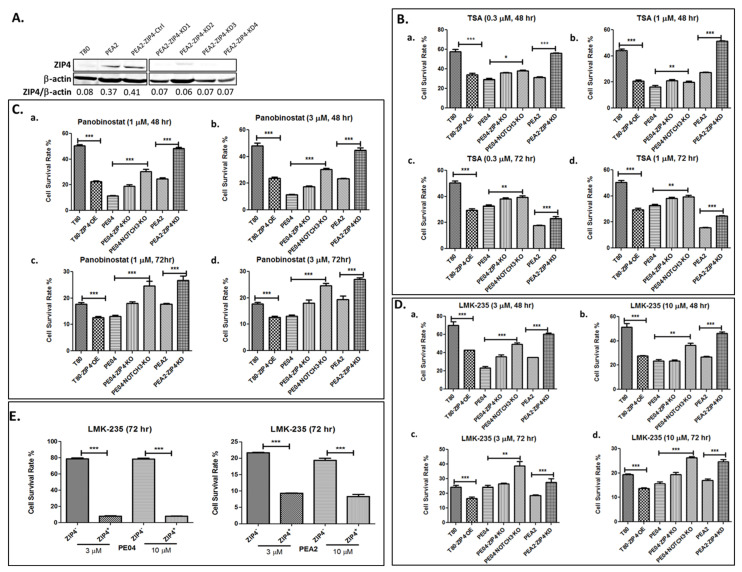
ZIP4 sensitized HDACi in HGSOC cells. (**A**). Established PEA2-ZIP4-KD cell lines. Other ZIP4-KO/KD, NOTCH3-KO cell lines used in this work were established and published previously [16,17]. The up- or downregulation of ZIP4 in these cell lines, including T80-ZIP4-OE, were confirmed and shown in Figure 2A. (**B**–**D**). ZIP4-OE in T80 (an immortalized human ovarian surface epithelial cell line) cells sensitized to TSA (0.3 and 1 µM), PANO (1 and 3 µM), and LMK-235 (3–10 µM). ZIP4-KO/KD in both PE04 and PEA2 cells resulted in more resistance to HDACis-induced cell death. (**E**). FACS-sorted ZIP4^+^ cells respond to LMK-235 (3 and 10 μM) significantly stronger than those ZIP4^−^ cells. The MTT values for each individual cell line with solvent treatment were used as 100% in all results shown in this figure. All experiments were repeated ≥ three times and presented as means ± standard deviation (SD). * *p* < 0.05; ** *p* < 0.01; *** *p* < 0.001.

We have previously shown that ZIP4 expression increased drug resistance to CDDP and doxorubicin [16,17]. To determine whether ZIP4 also affected the cellular response to other drugs in HGSOC cells, we tested additional pharmacologic inhibitors in pairs of ZIP4 differentially expressed cell lines: T80-vector control vs. T80-ZIP4-overexpression (OE) cells; PE04-vector control and PE04-ZIP4-KO cells; and PEA2-vector control and PEA2-ZIP4-KD cells (Figure 1A and published results for these established cell lines [16,17]). PE01/PE04 and PEA1/PEA2 cell line pairs were derived from two individual patients before (PE01 or PEA1) and after (PE04 or PEA2) the onset of multidrug resistance [88,89]. In addition, a PE04-NOTCH3-KO cell line was included, since we have shown that NOTCH3 is a functional downstream mediator of ZIP4 [17]. We found that neither ZIP4 KO/KD nor OE affected how HGSOC cells to respond to LY294002, a PI3K inhibitor or 5-Azacytidine, a DNA methyltransferase inhibitor in the concentration range (10^−10^ to 10^−6^ M) tested, suggesting ZIP4-induced drug-resistant is selective, not universal.

To our surprise, we found that ZIP4-OE significantly sensitized cells to trichostatin A (TSA), an HDACi. On the contrary, ZIP4-KO greatly increased cell resistance to TSA (Figure 1B). These effects were consistent in several pairs of ZIP4 differentially expressing cells (Figure 1B).

In addition to TSA, a pan-HDACi, we tested six FDA-approved HDACis: valproic acid (mainly targeting HDAC1), belinostat (a relatively weak pan-HDACi); mocetinostat (targeting HDAC1-3), panobinostat (PANO, a pan-HDACi), pracinostat (targeting HDAC1-11), and entinostat (targeting HDAC1,3). ZIP4 sensitized cells to other pan-HDACis, such as PANO and pracinostat, but not those Class I selective HDACis, such as valproic acid, mocetinostat, and entinostat (Figure 1C and Supplementary Figure S1). These results suggest that this ZIP4 effect is not Class I HDAC dependent. We then tested a HDAC4/5 (both of them belong to Class IIa HDACs) selective inhibitor LMK235 [90] and found similar ZIP4-dependent sensitization to LMK235 (Figure 1D), suggesting that this ZIP4 effect is Class II HDAC selective. We confirmed this ZIP4-dependent effect further using FACS-sorted ZIP4^+^ and ZIP4^−^ cells obtained as we previously described [17]. As shown in Figure 1E, ZIP4^+^ cells were much more sensitive to LMK235 than ZIP4^−^ cells.

### 2.2. ZIP4 Induced Upregulation of Class IIa HDACs

One of the potential mechanisms by which ZIP4 sensitizes HDACis is that ZIP4 upregulates HDACs to make cells more sensitive to their inhibitors. We tested ZIP4′s effect on representative HDACs in Class I (HDAC1, 2 and 3), Class IIa (HDAC4, 5, and 7), Class IIb (HDAC6 and 10) and Class IV (HDAC 11).

**Figure 2:**
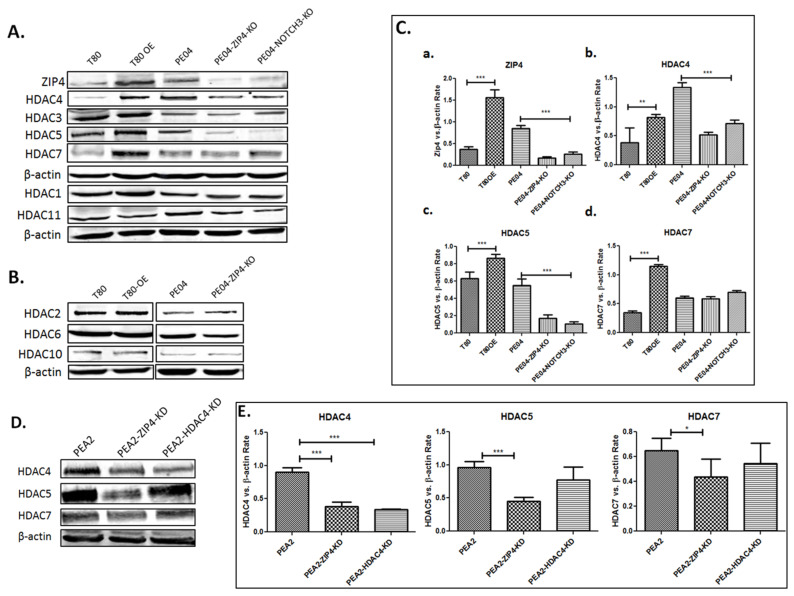
ZIP4 upregulated Class IIa HDACs. (**A**,**B**). ZIP4-OE up regulated the expression of HDAC4, 5, and 7; ZIP4-KO and NOTCH3-KO downregulated the expression HDAC4, 5, and 7 in PE04 cells, with little or no effect on other classes of HDACs. (**C**). Summary of quantified Western blot results from > three independent experiments for ZIP4, HDAC4, 5, and 7. (**D**). PEA2 cells expressed relatively higher levels of HDAC4, 5, and 7 than PEA1 cells; ZIP4-KD reduced expression levels of HDAC4, 5, and 7 in PEA2 cells. (**E**). Summary of quantified Western analyses results from > three independent experiments. * *p* < 0.05; ** *p* < 0, 01; *** *p* < 0.001.

ZIP4-OE significantly increased the expression of HDAC4 and 5 and ZIP4-KO significantly decreased the expression of HDAC4 and 5 as shown in Figure 2A, as well as in Figure 2C (quantitative data summarized from > three independent experiments for ZIP4, HDAC4,5, and 7). While ZIP4-OE significantly increased the expression of HDAC7, ZIP4-KO in PE04 cells did not significantly reduce HDAC7′s expression (Figure 2A,C). It is possible that the basal level of HDAC7 is not regulated by ZIP4 in PE04 cells. We also tested ZIP4′s effect on HDAC4, 5, and 7 and found that ZIP4-KD in PEA2 cells reduced their expression, with HDAC4-KD only affecting HDAC4 itself (Figure 2D,E). These results confirmed the regulatory effect of ZIP4 on Class IIa HDACs. In contrast to its effect on Class IIa HDACs, ZIP4 had minimal or no effect on HDACs in other classes, including Class I, Class IIb and Class IV (Figure 2A,B).

### 2.3. HDAC4 Played an Important Role in HDACi Sensitization in PE04 and PEA2 Cells

**Figure 3:**
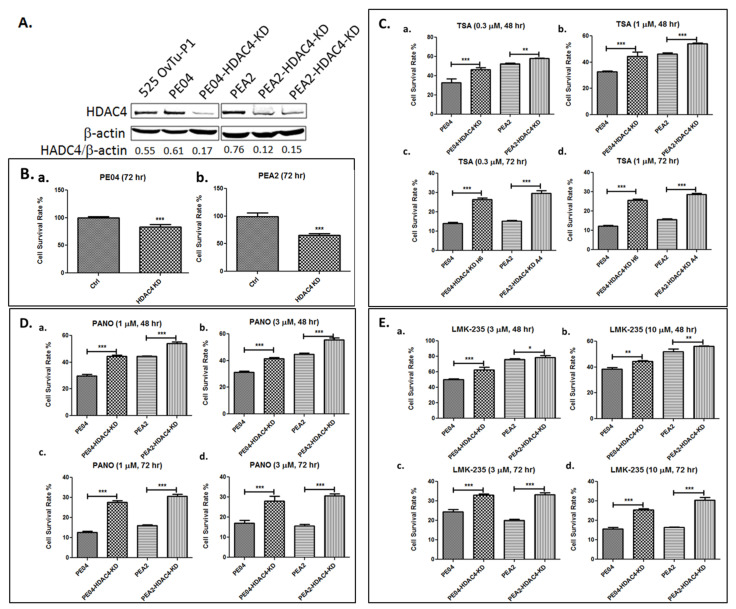
HDAC4-KD inhibited HDACi sensitization in PE04 and PEA2 cells. (**A**). HDAC4-KD PE04 and PEA2 cell lines generated using shRNAs. (**B**). HDAC4-KD PE04 and PEA2 cell lines reduced cell numbers (measured by MTT). (**C**–**E**). HDAC4-KD increased cell resistance to HDACis (TSA, PANO and LMK-235). The MTT values for each individual cell line with solvent treatment were used as 100% in (**B**–**D**). All experiments were repeated ≥ three times and are presented as means ± standard deviation (SD). * *p* < 0.05; ** *p* < 0.01; *** *p* < 0.001.

We decided to focus on HDAC4 for the rest of our studies due to its high clinical relevance as mentioned in the Introduction. To determine the role of HDAC4 in HDACi sensitization, we generated HDAC4-knockdown (KD) using shRNA in both PE04 and PEA2 cell lines (Figure 3A). HDAC4-KD reduced cell numbers (measured by MTT) by 17–35% (Figure 3B). Similar to ZIP4 KO/KD, HDAC4-KD cells increased cellular resistance to TSA, PANO, and LMK235 (Figure 3C–E), suggesting that HDAC4 plays an important role in cellular sensitivity to HDACis.

### 2.4. HDACis and HDAC4-KD Inhibited Spheroid Formation in HGSOC Cells

**Figure 4:**
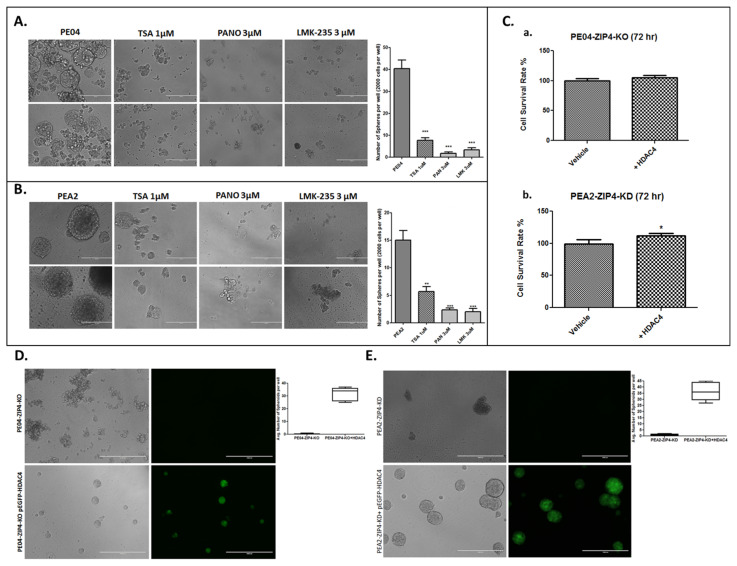
HDACis inhibited spheroid formation and HDAC4 gene compensated the spheroid formation activity in ZIP4-KO/KD cells. (**A**,**B**). HDACis (TSA, PANO, and LMK-235) inhibited spheroid formation in PE04 and PEA2 cells. The scale bars = 400 nm. Summary of quantified spheroid formation data from > three independent experiments in PE04 and PEA2 cells. (**C**)**.** HDAC4 gene transfected cells increased cell numbers (measured by MTT), compared to vector-transfected ZIP4-KO/KD cells. (**D**,**E**). HDAC4 gene transfected green cells compensated for the spheroid formation activity lost in ZIP4-KO/KD PE04 and PEA2 cells. The scale bars = 1000 nm. For quantification, cell aggregates < 70 nm and/or with irregular shapes were not counted as spheroids. Representative pictures are shown. * *p* < 0.05; ** *p* < 0.01; *** *p* < 0.001.

Spheroid formation is one of the standard assays for CSC [91,92,93]. ZIP4 was expressed in spheres, but not (or at lower levels) in nonsphere cells as we recently reported [17]. We tested the role of HDACis in both PE04 and PEA2 cells and found that TSA, PANO, and LMK135 dramatically inhibited spheroid formation in these cells (Figure 4A,B), suggesting that HDACis are potent CSC inhibitors.

We have shown that ZIP4-KO inhibited cell proliferation and spheroid formation in both PE04 and PEA2 cells [16,17]. To determine the functional relationship between ZIP4 and HDAC4, we then overexpressed the *HDAC4* gene with a plasmid co-expressing GFP in PE04-ZIP4-KO and PEA2-ZIP4-KD cells. HDAC4 overexpression (green cells) increased cell proliferation (Figure 4C) and restored the spheroid formation activity in ZIP4-KO/KD HGSOC cells (Figure 4D,E), confirming that HDAC4 is an important downstream mediator of ZIP4.

**Figure 5:**
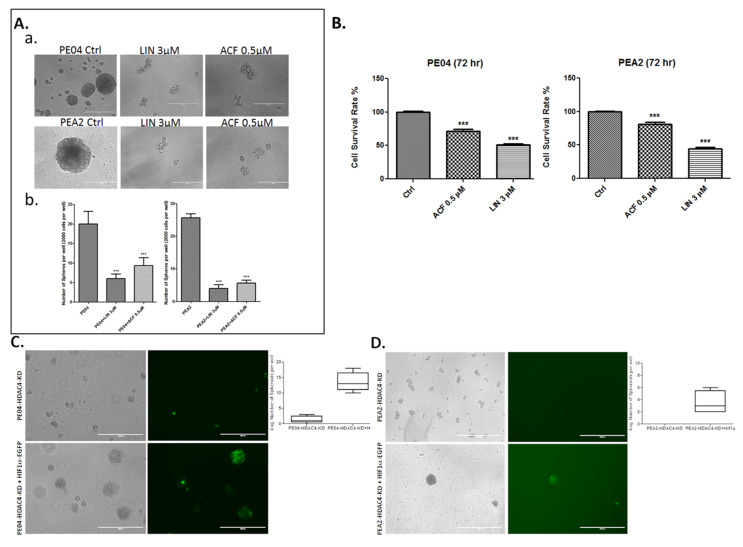

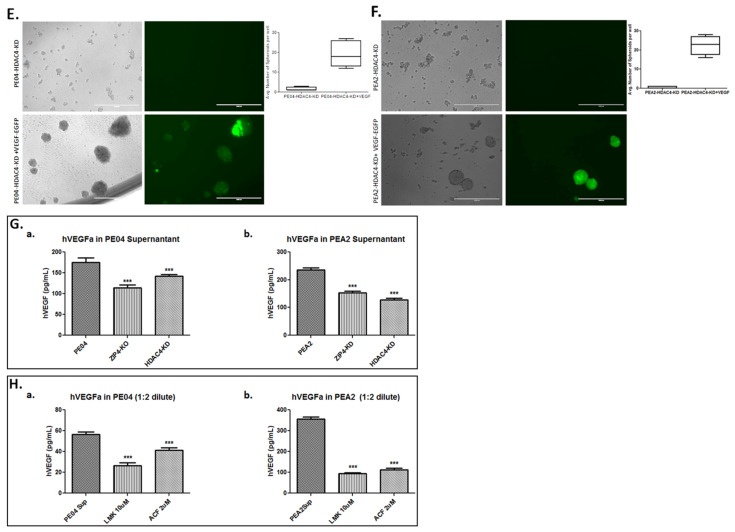
HIF1α and VEGFA were involved in spheroid formation and in regulating VEGFA secretion. (**A**,**B**). Linifanib (Lin, a selective inhibitor of VEGFA, 3 μM) and acriflavine (Acri, a selective inhibitor of HIF1α, 0.5 μM) inhibited cell proliferation and spheroid formation in PE04 cells. (**C**). Quantitative summary of the inhibitors’ effects from > three independent experiments. *** *p* < 0.001. (**D**,**E**). *HIF1α* gene transfected green cells compensated for the spheroid formation activity lost in HDAC4-KD PE04 and PEA2 cells. Representative pictures are shown. The scale bars = 1000 nm. (**F**,**G**). VEGFA gene transfected green cells compensated for the spheroid formation activity lost in HDAC4-KD PE04 and PEA2 cells. The scale bars = 1000 nm. (**G**). ZIP4-KO/KD and HDAC4-KD in PE04 and PEA2 cells reduced the secreted levels of VEGFA. (**H**). LMK235 and Acri inhibited secreted VEGFA in PE04 and PEA2 cells. For quantification, cell aggregates < 100 nm and/or with irregular shapes were not counted as spheroids. For quantification, cell aggregates < 70 nm and/or with irregular shapes were not counted as spheroids. Representative pictures from ≥ three independent experiments are shown. *** *p* < 0.001.

Cell proliferation measured in 2D cell culture and spheroid formation assays measured in 3D culture conditions detect partially overlapping (e.g., cell survival), yet different cellular properties. The latter reflects more CSC-related activities. We have shown previously that ZIP4-KO reduced cell numbers (measured by MTT) by 50–60% when compared to the parental cells (Figure 3 in [16]). HDAC4-KD reduced cell numbers by 17–35%, and HDAC4 overexpression increased cell numbers by 5–12%, when compared to the control cells (Figure 3B and Figure 4C). As shown in our previous publications [16,17] and in Figure 4 and Figure 5. ZIP4-KO and HDAC4-KD resulted in essentially complete blockage of spheroid formation. Comparison of the results from the 2D and 3D culture conditions suggest that both ZIP4 and HDAC4 play important roles in spheroid formation and CSC properties. Their effects on cell proliferation is unlikely to account for all of the lost spheroid formation activities.

### 2.5. The Role of the Hypoxia Inducible Factor-1 Alpha (HIF1α) and Endothelial Growth Factor A (VEGFA) in Spheroid Formation in HGSOC Cells

While many HDAC4 targets have been identified [47,94], we focused on HIF1α, one of the central players of tumor progression and drug response [47,48,49,50] and VEGFA, one of the best-characterized HIF1α targets [69,70,71]. Selective inhibitors for HIF1α and VEGFA, acriflavine and linifanib, respectively, inhibited cell proliferation and blocked spheroid formation in both PE04 and PEA2 cells (Figure 5A–C).

HDAC4-KD significantly inhibited spheroid formation (Figure 4C,D). To test the role of HIF1α or VEGFA in spheroid formation more specifically, we transfected *HIF1α* or *VEGFA* genes (co-expressing GFP) to HDAC4-KD PE04 and PEA2 cells. As shown in Figure 5C–F, only those cells transfected with either HIF1α or VEGFA (green cells) in HDAC4-KD cell lines formed spheroids, indicating that HIF1α and VEGFA are not only involved in CSC activity, but also are downstream mediators of HDAC4.

ZIP4-KD and HDAC4-KD in PEA2 cells reduced the levels of HIF1α (Figure S2). This may be related to HDAC4′s effect on HIF1α acetylation and stabilization as previously reported HIF1α [46,47]. In addition, we found that ZIP4/KD and HDAC4-KD in PE04 and PEA2 significantly reduced VEGFA production/secretion detected in cell supernatants (Figure 5G). LMK-235 and acriflavine significantly reduced VEGF production in PE04 and PEA2 cells (Figure 5H).

### 2.6. The Combinational Targeting CSC via the ZIP4-HDAC4 Axis and Non-CSC Using CDDP Was Highly Effective in Blocking Tumorigenesis

To test our overall hypothesis that co-targeting the ZIP4^+^ CSC, via ZIP4-KO/KD or HDAC4/KD, and the bulk ZIP4^−^ non-CSC (using CDDP) populations is crucial to develop more effective treatment modalities for blocking development of HGSOC, we used the PE04 xenograft mouse model and compared the tumor development and mouse survival times in five pairs of groups in the absence or presence of CDDP: PE04 control cells, PE04-ZIP4-KO cells, PE04-HDAC4-KD cells, PE04 control cells treated with PANO, and PE04 control cells treated with LMK235. The results are summarized in Table 1. In all mice, 5 × 10^6^ cells/mouse were i.p. injected with *n* = 5–6 in each group. Both genetic manipulations (such as ZIP4 or HDAC4 KO/KD) and HDACi (PANO or LMK-235) were mainly aimed at targeting ZIP4-dependent CSCs and CDDP were mainly aimed at targeting the bulk tumor cells.

**Figure 6:**
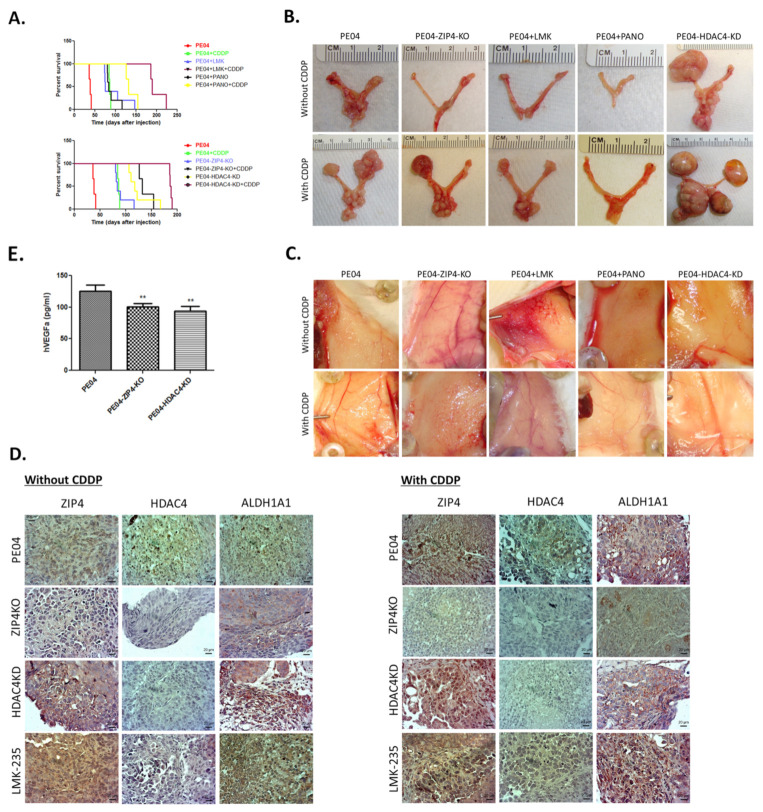
The combinational targeting of the ZIP4-HDAC4 axis and non-CSC using CDDP was highly effective in blocking tumorigenesis. (**A**) The survival curves from each group of mice, with the longest survival groups were those targeting HDAC4. (**B**) Representative tumors on the ovaries and the fallopian tubes from each group of mice. (**C**). Representative tumors on the peritoneal walls (PW) from each group of mice. Even though one or two of the five mice in the PANO and LMK-235 treated groups had low numbers of PW metastases (Table 1), most of them had none on the PW. (**D**). Representative ZIP4, HDAC4, and ALDH1 IHC staining. Magnification was 40×. (**E**). Human VEGFA in ascites in mice. Representative pictures from ≥ three independent experiments are shown. ** *p* < 0.01.

As shown in Table 1 and Figure 6A, genetically KO or KD ZIP4 and HDAC4, extended the mouse survival time from ~40 days to ~96 to 124 days, respectively. These data suggest that although ZIP4 was an upstream regulator of HDAC4, the latter is likely to be regulated by other factors and is likely to play a more important role in tumor development in vivo.

We found that single reagent treatments, including CDDP, PANO, and LMK-235 of PE04 cells, extended mouse survival days from ~40 days to 86–95 days (Table 1 and Figure 6A). At the endpoint, all three reagents reduced overall tumor volumes in mice, with LMK-235 being more effective. In addition, all three drugs reduced the volumes of ascites compared to the control group (Table 1).

The effects of combinational treatments with one arm on the ZIP4-HDAC4 axis and the other on CDDP were tested and compared. As expected, addition of CDDP significantly extended mouse survival times from the corresponding single reagent treatment group, by 85 (the ZIP4-KO group), 96 (the LMK235+CDDP group), 46 (the PANO+CDDP group), and 63 days (the HDAC4-KD) (Table 1 and Figure 6A), supporting the concept of the co-targeting strategy. The longest survival times were observed in the LMK-235+CDDP and the HDAC4-KD+CDDP groups (187–191 days), suggesting blocking HDAC4 is very critical and effective.

All of the drug treatments lasted for 4 weeks (starting 7 days after tumor cell injection). There was no drug delivered during the rest of the days when mice survived. While the mouse survival times were significantly extended with treatments, the tumors in long-lived mice grew back to even bigger sizes in some of the groups in a tissue location-specific manner (Table 1 and Figure 6C). For example, addition of CDDP increased tumor volumes on the ovaries and fallopian tubes as shown in the representative photos in Figure 6C. More dramatically, HDAC4-KD resulted in large tumors (10–15 mm in diameter) in the absence or presence of CDDP, around ovaries and fallopian tubes (Figure 6C), which was not observed in the LMK-235 groups, suggesting that there are differences between genetic depletion of the HDAC4 gene and pharmacological inhibition of HDAC4 and/or HDAC5. In addition, compared to control and ZIP4-KO mice, the mice in the groups treated with the two HDACis (PANO and LMK-235) and HDAC4-KD shared an interesting commonality: these reagents significantly blocked tumor metastases on the peritoneal walls (Figure 6D). These results suggest that HDAC4 may have an inhibitory role for large tumor development on the ovaries and fallopian tubes but facilitates peritoneal dissemination.

As shown in Figure 6D, these three CSC-related proteins were expressed in PE04 control cell-derived tumors. ZIP4-KO resulted in greatly diminished expression of ZIP4 and HDAC4 in tumor sections, with reduced ALDH1A expression. HDAC4-KD and LMK235, on the other hand, dramatically reduced HDAC4 expression, without significantly affecting ZIP4 and ALDH1A expression, supporting that ZIP4 is upstream of HDAC4. In general, when CDDP was added in combination with one of the ZIP4-HDAC4 axis modulators, it enhanced ZIP4, HDAC4, or ALDA1A expression in tumors from different mouse groups, supporting the idea that CDDP has the ability to induce CSCs [95]. We analyzed human VEGF in ascites and found that human VEGFA was downregulated in the PE04-ZIP4-KO and –HDAC4-KD groups when compared to the control PE04 group (Figure 6B).

**Figure 7:**
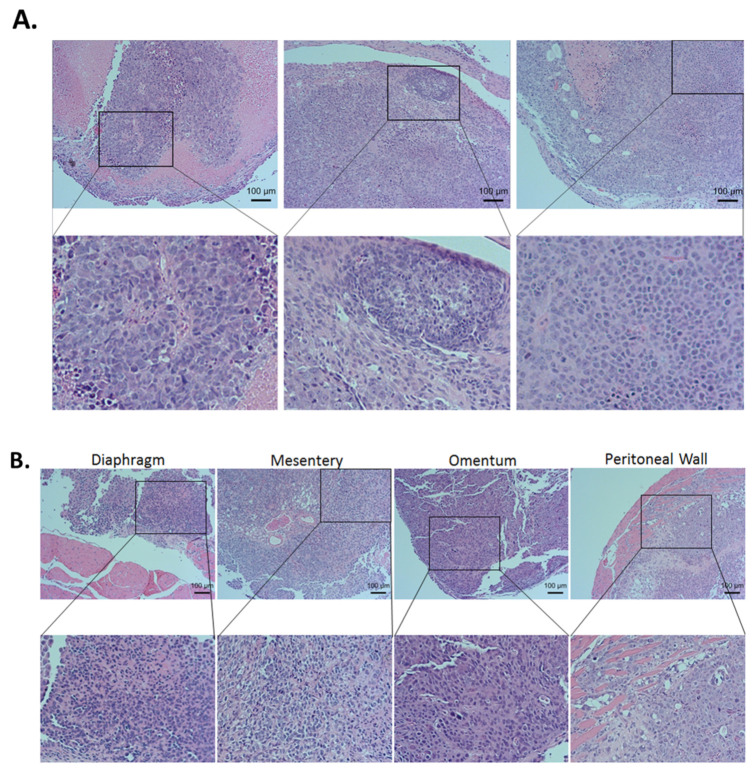
Representative H&E staining of the tumor sections from the ovaries (**A**) and representative H&E staining of the tumor sections from other organs (**B**).

The H&E staining of tumors from the ovaries (Figure 7A) or from other organs (Figure 7B) showed HGSOC histology confirmed by our pathologist. Immunohistochemistry (IHC) staining for ZIP4, HDAC4, and ALDA1A were conducted in tumors from different groups of mice.

### 2.7. ZIP4 and HDAC4 Were Upregulated in a Subset of Recurrent vs. Primary Human HGSOC Samples

**Figure 8:**
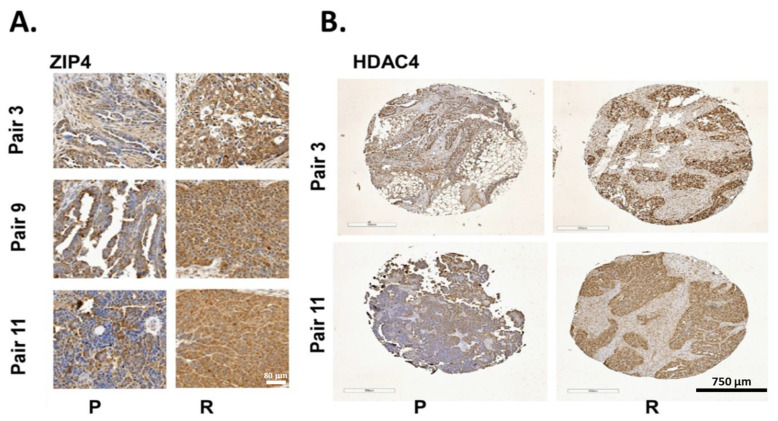
Co-overexpression of ZIP4 and HDAC4 in a subset of recurrent (R) vs. primary (P) human HGSOC. (**A**) ZIP4 was overexpressed in 3 of 10 recurrent (R) vs. primary (P) human HGSOC paired samples. (**B**). HDAC4 was overexpressed in 37% recurrent vs. primary paired samples tested, with two of these pairs also expressing high levels of ZIP4.

Our published data, along with TCGA and Oncomine data have shown and confirmed that ZIP4 is upregulated in human EOC tissues [13,16,96,97], justifying its clinical relevance to EOC. We tested ZIP4 and HDAC4 expressions in 10 pairs of primary and recurrent (platinum resistant) human HGSOC samples (each pair was from the same patient). Three of 10 these pairs overexpressed ZIP4 (more brown colored) in the recurrent vs. primary tumor tissues (Figure 8A). Among them, two of three of the ZIP4 overexpression pairs also had HDAC4 overexpression (Figure 8B). HDAC4 alone was overexpressed in 37% (7 of 19) of recurrent paired samples tested.

## 3. Discussion

Targeting CSC is a well-accepted concept for cancer treatment, especially for overcoming drug-resistance. Many studies have been conducted to target CSC in various cancers, including EOC [12,14]. Most of these studies focus on developing direct depleting or inhibiting the CSC marker. For example, several inhibitors have been developed and/or tested to inhibit ALDH isoforms in cancer CSC studies a very active field [9,98,99,100,101]. In addition, many of these studies have tested the effect of targeting CSC only (reviews [31,54,55,56,57] and references herein).

In the current work, however, we have challenged the current paradigm in developing anticancer strategies in several aspects. Firstly, CSCs have been considered mainly to display MDR in general. However, our data demonstrate that they may also exhibit high selectivity in drug response and sensitize to specific drugs. We developed and tested the strategy by taking the advantage of the preferential response to HDACis in ZIP4^+^ cells to selectively target CSCs, as a contrary to inhibit the CSC marker ZIP4 directly. We showed here that ZIP4^+^ CSCs were highly sensitive to HDACis, especially HDACis targeting IIa HDACs, providing feasible tools to target these cells selectively and efficiently. HDACis, and LMK-235 in particular, indeed displayed strong antitumor and anti-CSC activities. At the current stage, genetic manipulation is still relatively difficult to apply to humans directly and a selective inhibitor of ZIP4 has yet to be developed. On the contrary, several HDACis have been FDA approved and are in clinical trials.

Secondly, in the present work, we have tested our hypothesis that co-targeting CSC and non-CSC cell populations in cancer treatment is necessary and/or pivotally important for the treatment of EOC. While co-targeting the bulk and CSC populations in cancer have been reported (e.g., [57,58,102,103,104,105]), it is significantly underappreciated, partly due to a presumed and more rigid definition for CSCs, considering a cell population selected by a specific marker set is the only cell source initiating tumor formation. However, when compared to the developmental stem cell concept, CSC is more of an operational concept, mainly defined by their ability to initiate tumors and mainly identified by surface makers [8], with high translational/clinical application potential. The CSC phenotype varies substantially and may undergo reversible phenotypic changes. Non-CSCs can be induced into a stem-like state enabling them to become drug-tolerant [12,63,64,65,66,67,95]. Hence, it is important to co-target both subpopulations of tumor cells. In our in vivo mouse models, CDDP is used to target mainly the highly proliferative non-CSCs and inhibition of the novel ZIP4-HDAC4 axis is the major strategy for targeting CSCs. We used five sets of different mouse models to test and compare the efficacies: PE04 control cells ± CDDP; ZIP4-KO ± CDDP, HDAC4-KD ± CDDP, a pan-HDACi (PANO) ± CDDP, and a selective HDAC4/5 inhibitor, LMK-235 ± CDDP. In all five pairs of comparisons, even though single gene KO or KD or single HDACi significant increased mouse survival times, combination with CDDP clearly had significant additional benefit, extending additional mouse survival times ranging from 45 to 96 days (~4.5–9.6 human years [106]), supporting our overall hypothesis. It is important to note that CDDP treatment eventually enhanced expression of ZIP4, HDAC4, or ALDA1 in tumors from different mouse groups, supporting the idea that CDDP has the ability to induce CSCs [95]. These data suggest that a longer and continuous CSC-targeted treatment modality should be seriously considered.

Thirdly, although HDACis as a therapeutic strategy against CSCs has been studied using different CSC markers [107,108,109,110,111], the mechanisms have only been minimally or indirectly studied [112,113,114,115]. In addition, although combinational effects of an HDACi and CDDP in EOC cells have been tested (e.g., [36,60,61,62]) and reagents co-targeting CSC and non-CSC cell populations in cancer cell lines or mouse models have been reported (e.g., [57,58]), these studies did not test the effect in clearly defined CSC and non-CSC populations or were not linked to ZIP4. Importantly, most, if not all, of these studies were conducted in vitro and were focused on Class I HDACis. In fact, the majority of HDACi studies, including preclinical studies and clinical trials are mainly focused on Class I HDACs, since they are the main nuclear HDACs with high enzymatic activities and important physiological functions. However, in vivo HDACi toxicity observed up to date are also mainly caused by inhibiting HDAC1-3, the core nuclear histone deacetylase [116,117,118]. On the other hand, the concept that HDAC4 is a highly effective target is supported by the data presented here and it is not only more innovative, but also has the potential to significantly reduce toxicity. Comparison of our results from PANO and LMK235 suggests that developing more specific and selective HDACis is likely to be a promising direction to overcome the toxicity issues related to HDACIs.

Fourthly, we have previously shown that ZIP4 is an upstream regulator of several CSC markers, including ALDH1, OCT4, and SOX9 and NOTCH3 [16,17,119,120]. Here, we showed here that ZIP4 regulated HDAC4/5 and NOTCH3 may also be an upstream regulator of HDAC4/5, since NOTCH3-KO in PE04 cells also reduced the expression of HDAC4/5 (Figure 2). It is interesting to note that while ZIP4-KO extended mouse survival times from ~40 days to ~96 days, HDAC4-KD had an even longer survival time (~124 days). These data suggest that HDAC4 may also be regulated by other factors and may represent a better target for CSCs. In addition, HDAC4 may have organ-specific effects on tumor metastasis as shown in Table 1 and Figure 6. The larger, but more localized tumors resulting from HDAC4-KD may have clinical significance: these tumors may be easier to remove surgically than numerous microscopic metastases, a major source of recurrence. We also found that treatment with LMK-235, a selective inhibitor of HDAC4/5, did not result in large tumors in the ovaries and fallopian tubes, suggesting that this effect is more HDAC4-specific and/or not directly related to the inhibitory effect of LMK-235 on HDAC4. These concepts warrant further investigation in more HGSOC cell lines/PDXs.

Finally, it is almost certain that not all the functions of ZIP4 are mediated by HDAC4. We have already shown that ZIP4 also upregulates HDAC5, and possibly HDAC7 and/or HDAC9. The roles of HDAC5, 7 and 9 in CSC of HGSOC need to be further investigated. In addition, how ZIP4 regulates HDAC4 and other HDACIIa HDACs remains to be further investigated. Similarly, it is very likely that there are multiple downstream mediators of HDAC4, which play important roles in the overall effects of HDAC4 in cancer development in vivo. We focused on HIF1α and VEGFA in the current work, with other downstream mediators warranting further investigation. HGSOC is a highly heterogenic disease. Other than *TP53*, any other single gene occurs in 0.5 to 25% cases, with one exception (CDKN2A, 32%) [13]. Detecting 20–30% ZIP4-HDAC4 co-overexpression in human recurrent HGSOC (Figure 8) is highly significant to justifying the clinical significance.

## 4. Materials and Methods

### 4.1. Reagents, Cell Lines and Culture

For Western blot and immunohistochemistry (IHC) staining, and/or FACS assay and sorting, polyclonal ZIP4 antibody (20625-1-AP; Proteintech, Rosemont, IL, USA) was used. Anti-HDAC4 (ABIN6659710) antibody, anti-HDAC1 antibody (Catalog No. ABIN3020754), anti-HDAC2 antibody (Catalog No. ABIN3022867), anti-HDAC3 antibody (Catalog No. ABIN3023018), and anti-HDAC7 antibody (Catalog No. ABIN565634) were from Antibodies-Online Inc. (Limerick, PA, USA). Anti-HDAC4 antibody (A-4) (sc-46672), anti-HDAC6 (D-11) antibody (sc-28386), anti-HDAC10 (F-4) antibody (sc-376121), anti-HDAC11 (C-5) antibody (sc-390737) and anti-HIF-1alpha (28b) antibody (sc-13515) were from Santa Cruz Biotechnology (Beverly, MA, USA). HDAC5 (D1JV) Rabbit mAb (20458) was from Cell Signaling Technology, Inc. (Danvers, MA, USA).

The cancer cell lines used in this work were selected based on that (1) they are HGSOC cell lines; (2) the two pairs of cell lines were derived from two individual patients before (PE01 or PEA1) and after (PE04 or PEA2) the onset of multidrug resistance [88,89]; hence they are the most clinical relevant ovarian cancer cell lines to study drug resistance in EOC; and (3) we have recently shown that endogenous ZIP4 is expressed in all HGSOC cell lines examined, including PE04 and PEA2 cells, In contrast, several nontumorigenic and/or immortalized cell lines, including NIH3T3, Cos7, human ovarian surface epithelial (HOSE) cell lines, T29, T80, and a human fallopian tube cell line FT194 expressed low or undetectable ZIP4 (Figure 1, ref. [17]). In this work, we chose T80 for ZIP4 over-expression, since the endogenous ZIP4 expression in T80 cells was low. ZIP4 or HDAC4 genes were either knocked out using the CRISPR technology, knocked down using shRNA, or transfected with the human HDAC4 gene for overexpression, so that both gain- and loss-of-functional studies can be carried out. In addition, a PE04-NOTCH3-KO cell line was included, since we have shown that NOTCH3 is a functional downstream mediator of ZIP4 [17]. The pair of PE01/PE04 cell lines was from Dr. Daniela Matei (Northwestern University); the pairs of PEA1/PEA2 cells were from Sigma (St Louis, MO, USA). The T29 human ovarian surface epithelial cell line was from Dr. Jinsong Liu (M.D., Anderson). PE04-ZIP4-KO cell lines were generated using CRISPR as we described previously [16]. All cell lines were maintained in a humidified atmosphere at 37 °C with 5% CO_2_. PE01/PE04 cells were cultured in RPMI 1640 with glutamine, 10% fetal bovine serum (FBS), and 100 μg/mL Penicillin-Streptomycin-Amphotericin B. PEA1 and PEA2 were cultured in RPMI 1640 with 2 mM Glutamine, 2 mM sodium pyruvate, 10% FBS and 100 μg/mL penicillin incubation (37 °C, 5% CO_2_). After enzymatic disaggregation, cells were harvested for experiments. Human HGSOC tissues were obtained from CHTN as we described in our previous studies through Dr. Xu’s IRB [121].

Acriflavine (Catalog No: A8126) was from Sigma-Aldrich (St. Louis, MO, USA); Linifanib (Catalog No: HY-50751) was from MEDCHEMEXPRESS LLC (Monmouth Junction, NJ, USA). VALPROIC ACID (Catalog No: 2815100) was from Fisher Scientific Company LLC (Hanover Park, IL, USA); Entinostat (Catalog No: A8171-50) and Pracinostat (Catalog No: A4095-5) were from APEXBIO TECHNOLOGY LLC (HOUSTON, TX, USA); Mocetinostat (HY-12164) was from MEDCHEMEXPRESS LLC (Monmouth Junction, NJ, USA); TSA (Catalog No: S1045), LMK-235 (Catalog No: S7569), Belinostat (Catalog No:S1085), and panobinostat (PANO, LBH589; Catalog No: S1030) were from Selleck Chemicals LLC (Munich, Germany). The Duo Set of Human VEGFA (Catalog No: DY293B) and Mouse VEGF (Catalog No: DY493) were from R&D System (Minneapolis, MN, USA).

### 4.2. Western Blot Analysis

Western blot analyses were conducted using standard procedures and proteins were detected using primary antibodies and fluorescent secondary antibodies (IRDye 800CW-conjugated or IRDye 680-conjugated antispecies IgG, Li-Cor Biosciences, Lincoln, NE, USA) as we described previously [5].

### 4.3. Fluorescence-Activated Cell Sorting (FACS) of ZIP4^+^ Cells

FACS-based sorting and analysis of marker ZIP4^+^ cells was conducted using the BD FACSAria cell sorter system (Becton-Dickinson, Franklin Lakes, NJ, USA) and BD LSR Fortessa Analyser (BD Biosciences, San Jose, CA, USA), and data analyzed by FlowJo V10 (BD Biosciences, San Jose, CA, USA) as we described in [17].

### 4.4. DNA Transfection and Establishment of Stable Clones

pEGFP-HDAC4 plasmid (Catalog No: 45636), pg-HIF-1alpha-EGFP plasmid (Catalog No: 87204), and pCCLc-MNDU3-VEGFA-PGK-EGFP-WPRE plasmid (Catalog No: 89609) were from Addgene (Watertown, MA, USA). Plates (six-well) were seeded with 5 × 10^4^ cell/well in 2 mL media 24 h before transfection; cells were 80%–90% confluent and were transfected with plasmid DNA (2–10 μg/well) using Lipofectamine 2000 Reagent (11668-019, Life Technologies, Grand Island, NY, USA) according to manufacturer’s instruction. After 48 h of transfection, cells were seeded into ultralow attachment 24-well plate for sphere-formation assay.

Human HDAC4 shRNA (Catalog No: sc-35540-V) and ZIP4 shRNA (Catalog No: sc-77764-V) Lentiviral Particles were from Santa Cruz Biotechnology (Beverly, MA, USA). shRNA lentivirus vectors were co-transfected with packaging vectors to 293T cells in OptiMEM media. PE04 or PEA2 cells were infected by viruses three times and stable clones were selected by puromycin (0.5 μg/mL) for 10 days.

### 4.5. Spheroids-Formation Assays

As described in detail in our recent publication [17].

### 4.6. VEGFA ELISA Assays

Cells (1 × 10^4^/well) plated in six-well plates were grown in 2 mL of RPMI-1640 medium with 10% FBS for 24 h and then cultured in serum-free medium for 48 h. The culture supernatants were collected and their human VEGFA concentration were measured using ELISA kits (R&D). Ascitic fluids were collected from groups of treated and control female NSG mice (groups: PE04 with or without CDDP treatment; PANO with or without CDDP, LMK235 with or without CDDP; and HDAC4-KD with or without CDDP) and stored at −80 °C until ELISA assays were conducted. Ascites was diluted 8- to 16-fold in 1× reagent diluent for the ELISA assays. Both human VEGFA and mouse VEGF ELISA assays were performed using the DuoSet VEGFA ELISA kits from R&D Systems (Minneapolis, MN, USA) in triplicate wells according to the manufacturer’s instructions. The optical density at 450 nm was measured on an automated plate reader (PerkinElmer, Santa Clara, CA, USA). Experiments were repeated three times.

### 4.7. Immunohistochemistry (IHC) Staining

Anti-ZIP4 antibody (Proteintech) anti-HDAC4 (D15C3) antibody (Cell Signaling Technology), and anti-ALDH1A1 antibody (Sigma) were used as primary antibodies (1:100, 4 °C overnight). A VECTASTAIN^®^ Universal Quick HRP Kit (PK-7800) and a Vector NavaRED Substrate Kit Substrate Kit (SK-4800, Vector Laboratories, Burlingame, CA, USA) were used for detection. Tumor tissues from different groups of mice were examined in immunohistochemistry (IHC) staining. For IF Staining, Alexa Fluor antibodies (PE Donkey anti-Rabbit Cat.Log# 406421 and FITC Donkey anti-Goat Cat.Log# sc-3853) were used as secondary antibodies. Nuclei were counterstained using 4′, 6-diamidino-2-phenylindole (DAPI; Santa Cruz Biotechnology). The staining was analyzed using a Nikon Eclipse 80i microscope (Nikon, Badhoevedorp, The Netherlands).

### 4.8. HDACi Cytotoxicity Assay

Cell viability under HDACs inhibitions were performed using different concentrations of pan-HDAC inhibitors TSA and PANO, as well as LMK-235, a selective HDAC5/HDAC4 inhibitor. All of them were dissolved in 100% dimethyl sulfoxide (DMSO) according to the manufacturer’s protocol to a stock concentration of 20 mM and stored at −20 °C. Cells were seeded at a density of 2000 cells per well in 96-well plates. After culturing for 6 h, various doses of TSA (Sigma), panobinostat (PANO, VWR) or LMK-235 (VWR) were added. After cells were exposed to drugs for the indicated times (24/48/72/h), cell viability was determined using the MTT assay as described previously (81).

### 4.9. Xenograft Mouse Model

Female NSG mice were obtained from the In Vivo Therapeutics Core, Indiana University School of Medicine (Indianapolis, IN, USA)). For the characteristics assessment of chemotherapy resistant tumors and combined treatment efficacy in vivo, the genetically engineered PE04 cells [vector-transfected parental cells, ZIP4-knockout (KO), or HDAC4-knock down (KD)] (5 × 10^6^ cells) in 200 μL of DMEM/mouse] were intraperitoneally (i.p.) injected into 6- to 8-weeks old female NSG mice, Drug treatments started 14 days after tumor cell injection. Drugs were administered three times/week for 4 weeks, including controls [solvent, PANO (20 mg/kg), LMK-235 (13 mg/kg, i.P.; and/or CDDP (2.5 mg/kg)].

Engrafted mice were inspected daily for tumor/ascites appearance by visual observation, palpation, and tumor latencies. Mice were sacrificed by cervical dislocation at obvious distended abdomens or at >200 days post-transplantation. Tumors were counted at each metastatic location and tumor diameters were measured. Data are represented as mean ± SD (*n* = 5). The curves represented the trend of the surviving days increased after co-treatments. Animal protocols were approved by the Indiana University School of Medicine Animal Care and Use Committee (#11345). Xenograft tumors were resected, fixed in 10% neutral, buffered formalin, and embedded in paraffin for sectioning (5 μm) on a rotary microtome, followed by slide mounting, H&E staining, and histologic assessment by Dr. Robert Emerson, a pathologist at the Indiana University School of Medicine.

### 4.10. Human HGSOC IHC

Formalin-fixed and paraffin-embedded primary ovarian cancer tissues (all from HGSOC patients) were obtained from the Department of Pathology at the Johns Hopkins Hospital, Baltimore, Maryland. Specimens with tumor cell population > 50% and minimal or no necrosis were included in this study. Specimens were arranged in tissue microarrays to facilitate IHC and to ensure that the tissues were stained under the same conditions. The study was approved by the Johns Hopkins University School of Medicine Institutional Review Board. The scoring of IHC is the following method published [122].

### 4.11. Statistical Analyses

The Student’s *t*-test was utilized to assess the statistical significance of the difference between two treatments. The asterisk rating system as well as quoting the *p* value in this study was * *p* < 0.05; ** *p* < 0.01; and *** *p* < 0.001. A *p* value of less than 0.05 was considered significant.

## 5. Conclusions

Our studies have shown that co-targeting the ZIP4-HDAC4-VEGFA axis with a common chemotherapy represents new strategies to circumvent drug resistance in vitro and in vivo for HGSOC, a deadly disease. These studies significantly expand our understanding of the roles, the molecular mechanisms, and interactions of ZIP4 with other signaling molecules, as well as its potential clinical targeting value in EOC. 

## Figures and Tables

**Table 1:** Summary of tumor and ascites formation in different mouse groups.

Group	Cell Type	Cell No.	Treatment	Mice No.	Survival Days	Ascites Vol. (mL)	Tumors ≤ 1 mm	Tumors 1–5 mm	Tumors 5–10 mm	Tumors 10–15 mm	Tumor Sites
PE04	PE04	5 × 10^6^	None	3/3	40 ± 6	13 ± 2	36.6 ± 4.5	12 ± 2.6	2.3 ± 2	0	Ov, Pw, D, Om, M, P, SI
PE04 + CDDP	PE04	5 × 10^6^	CDDP (2.5 mg/kg)	4/4	86 ± 3	4.7 ± 1.1	23.8 ± 4.5	11.8 ± 4.8	0	0	Ov, Pw, P, Om, M
PE04-ZIP4-KO	PE04-ZIP4-KO	5 × 10^6^	None	5/5	96 ± 8	3.5 ± 0.7	15 ± 2	7 ± 2	0	0	Ov, Pw, D, M
PE04-ZIP4-KO+CDDP	PE04-ZIP4-KO	5 × 10^6^	CDDP (2.5 mg/kg)	4/4	141 ± 17	7.8 ± 2.7	22.2 ± 5.9	13.6 ± 5.9	0	0	Ov, Pw, SI, Om, D, M
PE04-PANO	PE04	5 × 10^6^	Panobinostat (20 mg/kg)	5/5	90 ± 15	9 ± 3.7	20.7 ± 14.9	10 ± 7.7	2.5 ± 2	0	Pw(1/5), D, M, P, SI
PE04-PANO +CDDP	PE04	5 × 10^6^	CDDP (2.5 mg/kg) +Panobinostat (20 mg/kg)	5/5	136 ± 11	0.6 ± 0	6 ± 3.5	0.4 ± 0.2	0	0	Pw (2/5), D, P
PE04-LMK	PE04	5 × 10^6^	LMK-235 (13 mg/kg)	5/5	95 ± 32	8.2 ± 3.8	13.8 ± 3.4	1.4 ± 1	0	0	Pw (2/5), D, M, Panc
PE04-LMK+CDDP	PE04	5 × 10^6^	CDDP (2.5 mg/kg) + LMK-235 (13 mg/kg)	5/5	191 ± 19	4.2 ± 4	13 ± 7.5	3.8 ± 3	0.5 ± 0.1	0	Ov, Pw (1/5), Om, P
PE04-HDAC4-KD	PE04-HDAC4-KD	5 × 10^6^	None	5/5	124 ± 25	7.1 ± 2.6	23.4 ± 11.1	13.6 ± 7.1	2.2 ± 1.9	1.2 ± 1	Ov, P, D, Om, Sp, SI, L
PE04-HDAC4-KD+CDDP	PE04-HDAC4-KD	5 × 10^6^	CDDP (2.5 mg/kg)	5/5	187 ± 3	3.1 ± 1.4	4.7 ± 2.8	2.2 ± 2	3 ± 1.5	4 ± 1.2	Ov, L, P, D, Om

Different PE04 cells, the type of treatments, mouse survival days, tumor and ascites volumes, and tumor locations are summarized. D, diaphragm; L, liver; M, mesentery; Om, omentum; Ov, ovary; P, pancreas; PW, peritoneal wall; SI, small intestine; Sp, spleen.

## Data Availability

No additional data.

## Supplementary Materials

The following are available online at https://www.mdpi.com/article/10.3390/cancers13153821/s1, Figure S1: ZIP4 only sensitized Class IIa targeting HDACi in HGSOC cells, Figure S2: The effects of ZIP4 and HDAC4 on HIF1α expression in PEA2 cells.
