# Supplementary material for: A Novel ZIP4-HDAC4-VEGFA Axis in High-Grade Serous Ovarian Cancer
Source: Cancers (Basel). 2021 Jul 29;13(15):3821. doi: 10.3390/cancers13153821 (PMC8345154; doi:10.3390/cancers13153821)
Supplement: Supplementary file 1 [file cancers-13-03821-s001.zip › cancers-1319192-supplementary.pdf]

Supplementary Materials

ZIP4 sensitized HGSOC cells to ClassIIa HDACis.

In addition to TSA, a pan HDACi, we tested six FDA-approved HDACis: valproic acid (mainly targeting HDAC1), belinostat (a relatively weak pan HDACi); mocetinostat (targeting HDAC1-3), panobinostat (PANO, a pan HDACi), pracinostat (targeting HDAC1-11), and entinostat (targeting HDAC1,3). ZIP4 sensitized cells to other pan HDACis, such as PANO and pracinostat , but not those Class I selective HDACis, such as valproic acid, mocetinostat, and entinostat (**Fig. S1**). These results suggest that this ZIP4 effect is not Class I HDAC dependent.

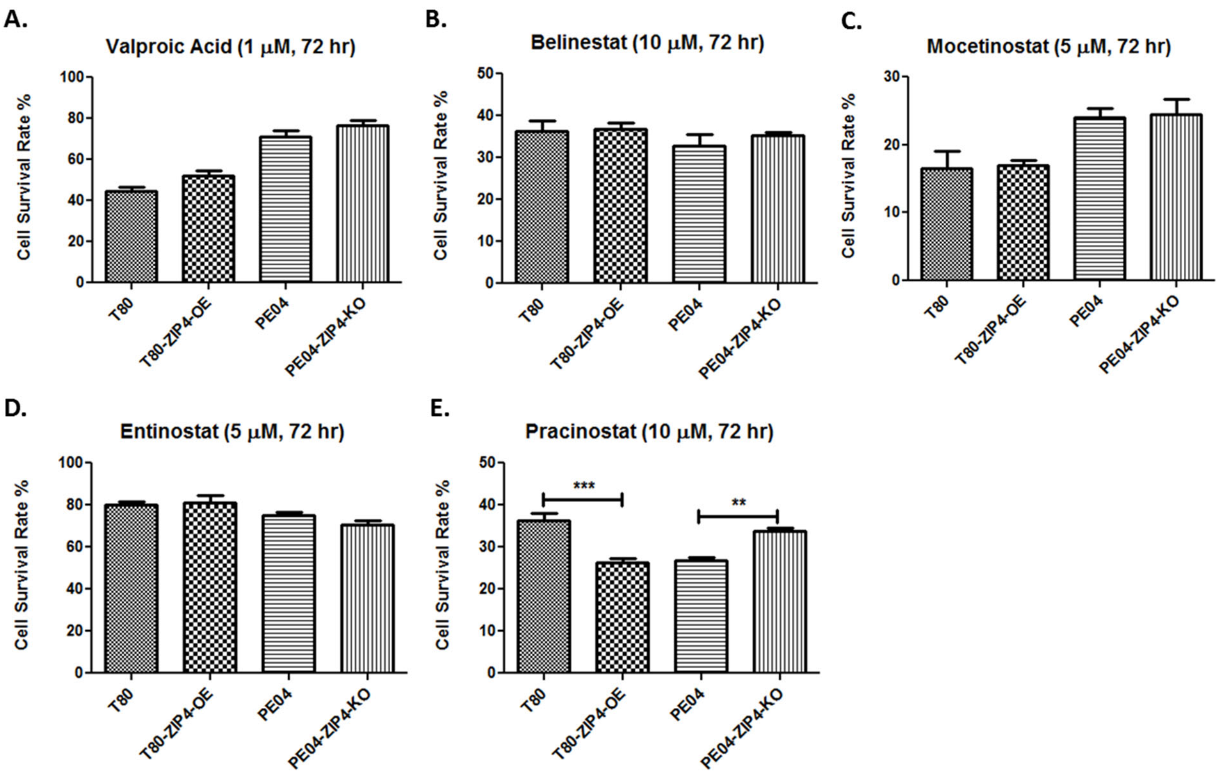

Figure S1. ZIP4 only sensitized Class IIa targeting HDACi in HGSOC cells**.** ZIP4-OE in T80 cells, ZIP4 or NOTCH3-KO in PE04 cells did not significantly change cell proliferation/survival responses to different valproic acid (**A**), belinostat (**B**), mocetinostat (**C**), and entinostat (**D**). In contrast, ZIP4-OE in T80 cells sensitized to pracinostat and ZIP4- and NOTCH3-KO in PE04 cells resulted in more resistance to pracinostat-induced cell death (**E**).

The effects of ZIP4 and HDAC4 on HIF expression

Under normoxic conditions, we found that ZIP4/KD and HDAC4-KD in PEA2 cells reduced the levels of HIF1α (Figure S2). This may be related to HDAC4's effect on HIF1α acetylation and stabilization as previously reported HIF1α [47, 48].

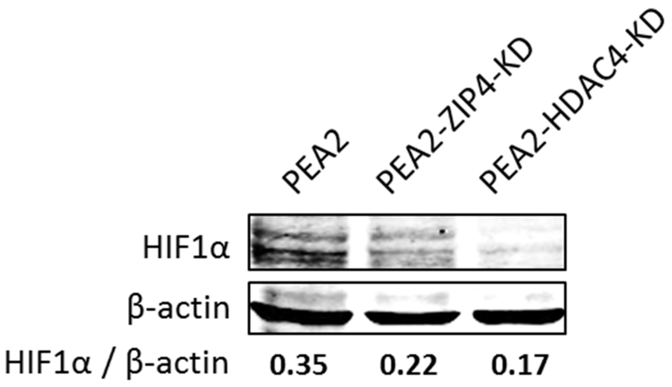

Figure S2. The effects of ZIP4 and HDAC4 on HIF1α expression in PEA2 cells. The antibody used was a purified Mouse Anti-Human HIF1α antibody (Cat Log#: 610958, BD Biosciences, San Jose, CA, USA).
